# Generalization in *de novo* learning of virtual upper limb movements is influenced by motor exploration

**DOI:** 10.3389/fspor.2024.1370621

**Published:** 2024-03-06

**Authors:** Tomoya Kawano, Motoki Kouzaki, Shota Hagio

**Affiliations:** ^1^ Laboratory of Motor Control and Learning, Graduate School of Human and Environmental Studies, Kyoto University, Kyoto, Japan; ^2^ Laboratory of Neurophysiology, Graduate School of Human and Environmental Studies, Kyoto University, Kyoto, Japan; ^3^ Unit of Synergetic Studies for Space, Kyoto University, Kyoto, Japan

**Keywords:** sensorimotor transformation, visuomotor task, reaching movement, movement variability, joystick control

## Abstract

The acquisition of new motor skills from scratch, also known as *de novo* learning, is an essential aspect of motor development. In *de novo* learning, the ability to generalize skills acquired under one condition to others is crucial because of the inherently limited range of motor experiences available for learning. However, the presence of generalization in *de novo* learning and its influencing factors remain unclear. This study aimed to elucidate the generalization of *de novo* motor learning by examining the motor exploration process, which is the accumulation of motor experiences. To this end, we manipulated the exploration process during practice by changing the target shape using either a small circular target or a bar-shaped target. Our findings demonstrated that the amount of learning during practice was generalized across different conditions. Furthermore, the extent of generalization is influenced by movement variability in the control space, which is irrelevant to the task, rather than the target shapes themselves. These results confirmed the occurrence of generalization in *de novo* learning and suggest that the exploration process within the control space plays a significant role in facilitating this generalization.

## 1 Introduction

New motor skills are acquired when starting a new activity. For instance, beginner tennis players improve their abilities through consistent practice over time. In motor-skill acquisition, a variety of practices are crucial for motor exploration (1). For example, in the context of mastering tennis, precise targeting of various areas of the court and executing shots from different locations are perfected through many rounds of practice. Previous studies have demonstrated that the outcome of motor learning depends on the motor exploration process associated with the learning methods (1–3). However, the effect of the motor exploration process on the acquisition of new motor skills from scratch remains unclear.

The process of acquiring entirely new motor skills, referred to as *de novo* learning, involves constructing a new controller that implements a novel sensorimotor transformation (4–6). De novo learning has been studied using tasks that require participants to learn new mapping of their body position to the location of an on-screen cursor (7, 8). This learning process differs from motor adaptation, which refers to the ability to adjust an existing controller to adapt to a changing environment (9–11). In adaptation tasks, when participants practiced one condition, the amount of learning was generalized to other conditions (12, 13). The motor experience available for learning is inherently limited when acquiring new motor skills from scratch. Consequently, in *de novo* learning, the ability to generalize the skills acquired for one condition to new conditions plays a crucial role in facilitating learning. In addition, previous studies have demonstrated that in force field adaptation, a generalization pattern is formed around actual rather than planned movements (14, 15). These results suggest that the motor exploration process, that is, how motor experience accumulates, is important for the generalization of learning. However, the relationship between the exploration process during *de novo* learning and generalization remains unclear.

Motor variability plays the role of motor exploration, which is necessary to learn a new motor skill (16). Several studies have reported that motor variability in the control space facilitates motor learning (17–20). In redundant motor tasks, the control space is comprised of two subspaces: task-relevant and task-irrelevant space (21, 22). To minimize motor errors during the learning process, motor commands must be modified in a task-relevant space (23). Therefore, the motor system must explore the motor commands and identify the task-relevant space. Moreover, a previous study theoretically demonstrated that motor exploration in a task-irrelevant space facilitates *de novo* learning (23). Therefore, in this study, we attempted to manipulate the exploratory process during *de novo* learning.

Previous studies demonstrated that the control strategy during upper limb reaching movements varied depending on the shape of the target (24, 25). For instance, when reaching toward a small circular target is laterally perturbed, corrective movements are elicited, directing the movement towards the location of the target. In contrast, when perturbations are introduced while reaching toward a larger bar-shaped target, movement trajectories are redirected to different nearby locations along the bar axis (25). Therefore, we exploited these distinct control strategies depending on task goals to manipulate the exploration process during *de novo* learning. It is expected that when learning with a small target, motor exploration characterized by corrective movements will be elicited, whereas when learning with a wide target, motor exploration involving various reaching directions will be predominant. We compared skill acquisition through these two exploratory processes and investigated the factors affecting generalization in *de novo* learning.

This study aimed to clarify how the exploration process in *de novo* learning affects the generalization of acquired skills. We designed a *de novo* visuomotor learning task with two different exploration processes by changing the shape of the target during the training phase. Following training, we assessed the ability of the two exploration processes to broadly and accurately apply the newly acquired skills.

## 2 Materials and methods

### 2.1 Participants

Thirty-two right-handed participants without a history of neurological or motor disorders participated in this study (28 males and 4 females; aged 22.3 ± 2.06 years, mean ± standard deviation). They provided informed consent after receiving a detailed description of the purpose, potential benefits, and risks of the experiment. All procedures used in this study were performed in accordance with the Declaration of Helsinki and were approved by the Ethics Committee for Human Experimentation at the Graduate School of Human and Environmental Studies, Kyoto University (22-H-21).

### 2.2 Experimental apparatus

**Figure 1:**
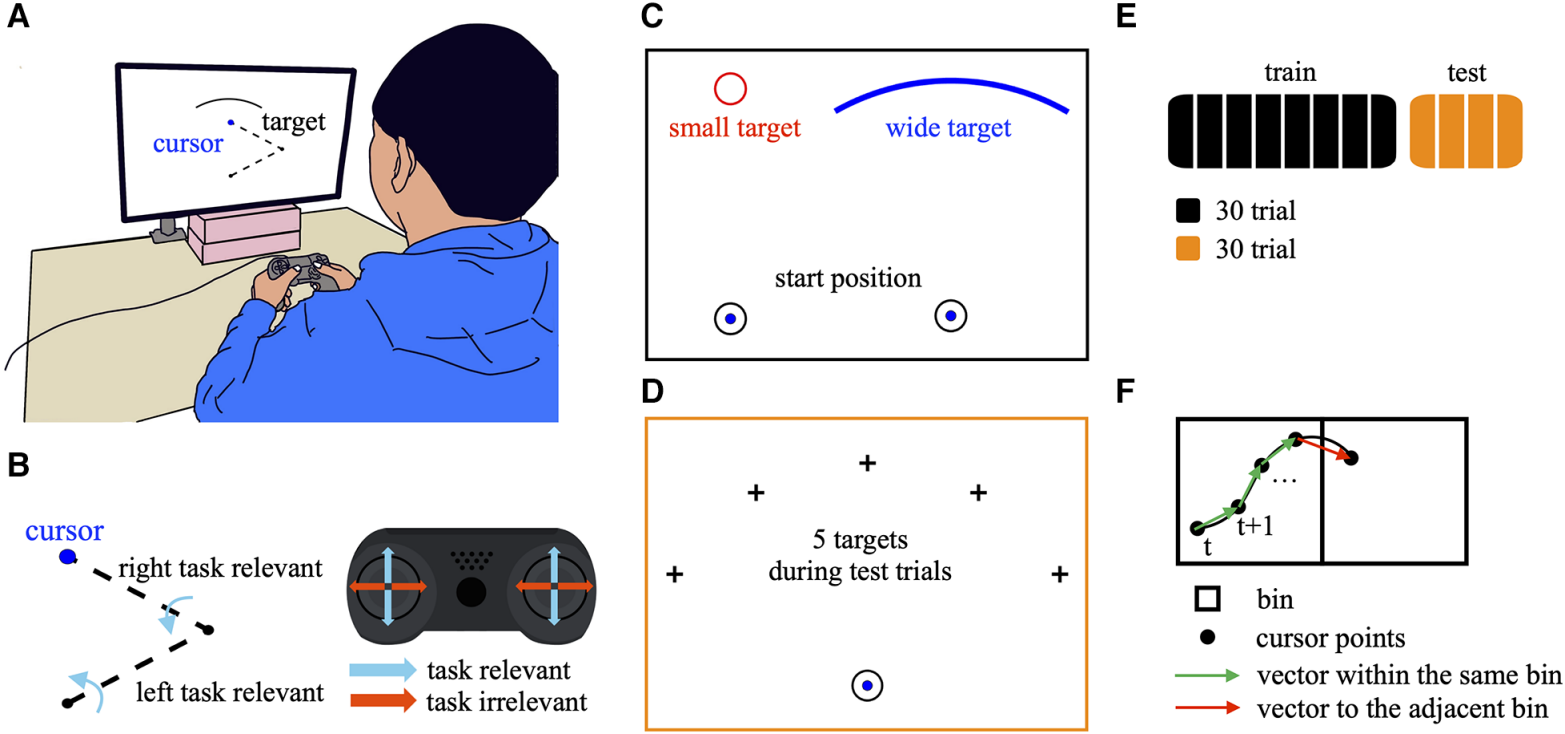
Experimental design. (**A**) Participants manipulated two joysticks on the gamepad using both thumbs to control the onscreen cursor. (**B**) Cursor position corresponding to the tip of the virtual two-link arm in the frontal plane. The up/down inputs from the right and left joysticks determined the torque applied to the proximal and distal joints, respectively. Therefore, the up/down inputs were directly relevant to the cursor movements (task-relevant). Conversely, the left/right inputs from both joysticks were irrelevant to the cursor movements (task-irrelevant). (**C**) In the training task, the participants were required to quickly move the cursor from the starting position to the target. The participants were divided into two groups based on the target shapes: small and wide. In the small target condition, the target was a small circle with a 10 mm diameter, whereas in the wide target condition, the target was an arched and wide shape with a curvature radius of 68 mm, width of 79 mm, and center angle of 60°. (**D**) The test task involved moving the cursor towards a target selected pseudo-randomly from 1 of 5 directions: −60°, −30°, 0° (above the starting position), 30°, 60°. (**E**) Sequence of tasks across the training and test phases. During the training phase, the participants performed 240 trials divided into eight blocks (30 trials per block). During the test phase, the participants performed 120 trials divided into four blocks (30 trials per block). (**F**) Quantification of the movement direction. The black square represents a bin. The black circles represent cursor points in a trajectory. The green arrow indicates a vector within the same bin. The red arrow indicates a vector in the adjacent bin.

The participants were seated in front of a gamepad (DUALSHOCK 4, Sony Interactive Entertainment Inc., Japan) placed on a table equipped with a 27-inch LCD monitor (Figure 1A). Participants manipulated the two joysticks on the gamepad using both thumbs. A cursor with a 4 mm diameter was displayed on the monitor. The cursor position corresponded to the tip of the virtual two-link arm in the frontal plane (Figure 1B). The dynamics of the two-link arm are as follows.

$$\begin{array}{rl} & \tau =I(\theta )\ddot{\theta }+B(\theta ,\dot{\theta })+V(\dot{\theta })\\ & I(\theta )=\left(\begin{array}{cc} I_{1}+I_{2}+m_{1}r_{1}^{2}+m_{2}r_{2}^{2} & I_{2}+m_{2}r_{2}^{2}\\ +m_{2}l_{1}^{2}+2m_{2}l_{1}r_{2}\cos\theta_{2} & +m_{2}l_{1}r_{2}\cos\theta_{2}\\ I_{2}+m_{2}r_{2}^{2}+m_{2}l_{1}r_{2}\cos\theta_{2} & I_{2}+m_{2}r_{2}^{2} \end{array}\right)\\ & B(\theta ,\dot{\theta })=\left(\begin{array}{c} -m_{2}l_{2}r_{2}(2{\dot{\theta }}_{1}+{\dot{\theta }}_{2}){\dot{\theta }}_{2}\sin\theta_{2}\\ m_{2}l_{1}r_{2}{\dot{\theta }}_{1}^{2}\sin\theta_{2} \end{array}\right)\\ & V(\dot{\theta })=\mu \dot{\theta } \end{array}$$

where *m_i_* = 4 [kg], *I_i_* = 0.27 [kg·m^2^], *r_i_*_ _= 0.15 [m], *ℓ_i_*_ _= 2*r_i_*_,_ and *µ* = 3 [N·m·s/rad] represent the mass, moment of inertia, the distance from the proximal joint to the center of mass, and the length of the proximal (*i* = 1), distal (*i* = 2) segments and coefficient of viscosity, respectively. The cursor movements displayed on the monitor were determined by the positions of the two joysticks on the two axes (X- and Y-axes), which were acquired in 16 bit resolution. The acquired joystick inputs were converted into torques, ***τ ***= (*τ_1_*, *τ_2_*)^T^, for each joint, ***θ*** = (*θ_1_*, *θ_2_*)^T^. The joystick provided two-dimensional inputs corresponding to the left/right and up/down directions from one side, resulting in four-dimensional inputs from both sides. However, only the up/down inputs from both joysticks were used to control the torque, as follows:

$$\left(\begin{array}{c} \tau_{1}\\ \tau_{2} \end{array}\right)=\left(\begin{array}{cc} \begin{array}{cc} 0 & a\\ 0 & 0 \end{array} & \begin{array}{cc} 0 & 0\\ 0 & a \end{array} \end{array}\right)\left(\begin{array}{c} s_{rx}\\ \begin{array}{c} s_{ry}\\ \begin{array}{c} s_{lx}\\ s_{ly} \end{array} \end{array} \end{array}\right)$$

where *a* (= 10^−4^) is the coefficient used to convert the joystick position to the virtual joint torque. The up/down inputs from the right joystick, *s_ry_*, and the left joystick, *s_ly_*, determined the torque applied to the proximal and distal joints and were thus directly relevant to the cursor movements (task-relevant). Conversely, the left/right inputs from the right and left joysticks, *s_rx_* and *s_lx_*, were irrelevant to the cursor movements (task-irrelevant). Only the position of the tip of the two-link arm was visible to the participants, with the links and joints not visible, ensuring that the participants did not explicitly realize how the cursor was moving.

### 2.3 Experimental procedure

The experimental task was to manipulate the cursor quickly from its starting position to the target. The starting position had a diameter of 10 mm and was located 15 mm above the center of the workspace (the proximal joint of the virtual two-link arm was located at the center). The target position was 68 mm above the starting position. This study consisted of two phases: the training and test phase.

In the training phase, participants were divided into two groups: small target condition (16 participants) and wide target condition (16 participants). These two conditions differed in target shapes (Figure 1C). In the small target condition, the target shape was a small circle with a diameter of 10 mm, whereas in the wide target condition, the target had an arched and wide shape with a curvature radius of 68 mm, width of 79 mm, and center angle of 60°. In the small target condition, the target position was only above the starting position (0° direction in the test phase; see below) throughout the training phase. The experimental procedures were identical, except for the target shape. After stopping at the starting position for 1 s, the target appeared and the participants were allowed to manipulate the cursor. When the cursor contacted the target, the target color changed from black to blue, and feedback on the movement duration was provided. And at that moment, the cursor automatically returned to the starting position. Therefore, participants could shoot the cursor, and were not required to stop the cursor on the target. This sequence constituted one trial and was repeated. The participants were instructed to reach the target as quickly as possible. In the wide condition, although the target was wide, no specific instructions were provided regarding where to aim. A total of 240 trials were completed and divided into 8 blocks (30 trials per block) during the training phase (Figure 1E).

The test phase was completed after the training phase. The task involved manipulating the cursor toward a target selected pseudo-randomly from 1 of 5 directions: −60°, −30°, 0° (above the starting position), 30°, 60° (Figure 1D). If the movement duration was less than 2.3 s, the trial was considered successful. Feedback on success or failure was provided based on the color of the target (green for success and red for failure). The movement duration and the 2.3-second criteria were not provided to participants. The participants were instructed to aim for as many successful trials as possible. A total of 120 trials were completed and divided into 4 blocks (30 trials per block) during the test phase (Figure 1E). Each target position was presented in six trials per block.

The experimental task was performed using a custom LabVIEW program (National Instruments Corp., Austin, TX, USA). Data, including the time sequence of the joystick input, cursor position, joint angle, and angular velocity of the two-link arm, were acquired at a sampling rate of 100 Hz.

### 2.4 Data analysis

All data were analyzed using MATLAB R2023a (MathWorks, Inc., Natick, MA, USA) and R version 4.2.2 (RStudio, Inc., Boston, MA, USA). The experiment involved 32 participants; however, data from one participant in the wide-target condition during the training phase were lost. Therefore, the training phase was analyzed using data from 31 participants.

To quantify task performance during the training phase, the movement duration was calculated as the time elapsed between the initiation of the task (when the target appeared) and termination (when the cursor contacted the target). Tortuosity, a measure of path straightness, was calculated as the total path length divided by the distance between the start and end positions of the cursor movement (26). A tortuosity value closer to 1 indicates a straight cursor trajectory.

The spatial exploration during the training phase as the directional variability of the cursor in the workspace was quantified (26). The following analysis was conducted on the cursor trajectory data for each block during the training phase. First, the workspace was divided into 39 × 39 bins, each measuring 50 × 50 mm. The coordinates of the cursor points for each trajectory were assigned to the corresponding bin. The cursor position at time *t* was defined as $p_{\left(x,y\right)}^{t}$, where *x* and *y* represented bin numbers ranging from 1 to 39. A vector from $p_{\left(x,y\right)}^{t}$ to $p_{\left(x,y\right)}^{t+1}$ was calculated for every time point and assigned to the bin corresponding to the vector starting point, considering cursor movements within the same bin and adjacent bins (Figure 1F). This process was repeated each time the cursor passed through a bin across all the trajectories in each block. Bins with fewer than three vectors were excluded from analysis. The mean vector angle and angular standard deviation were calculated by circular statistics to measure the average and variability of the movement direction for each bin. Movement direction variability (MV) was determined as the weighted average of the angular standard deviations across all bins, with weights corresponding to the number of vectors per bin. This analysis was applied to trajectory data from all blocks during the training phase. In addition, the reach direction was computed as the angle of the vector from the start to the final reach positions in each trial. Reach direction variability (RV) was calculated by circular statistics as the standard deviation of the reach directions across trials in each block. These two variability indices, MV and RV, were compared between different training conditions.

In this task, the participants were required to explore the joystick movement in task-relevant rather than task-irrelevant directions. Therefore, the task-irrelevant variability of the joystick input position was estimated as the index of the control space exploration during the training phase. For each block, the 4-dimensional average vector of the joystick input position for all trials, $\bar{s}=[{\bar{s}}_{rx},\;{\bar{s}}_{ry},\;{\bar{s}}_{lx},\;{\bar{s}}_{ly}{]}^{T}$, was calculated. Task irrelevant variability, *T_irr_*, was calculated as the mean squared distance between the average task-irrelevant components of $\bar{s}$ (i.e., $[{\bar{s}}_{rx},\;{\bar{s}}_{lx}{]}^{T}$) and task-irrelevant inputs ([*s_rx_, s_lx_*]^T^), according to the equation:

$$T_{irr}=\;\frac{1}{T}\overset{T}{\underset{t=1}{\sum }}\{{\left(s_{rx,t}-\;{\bar{s}}_{rx}\right)}^{2}+{\left(s_{lx,t}-\;{\bar{s}}_{lx}\right)}^{2}\}$$

where *T* represents the number of data points in all the trials in one block. Low task-irrelevant variability indicates that the joystick was controlled in task-relevant directions.

In test trials, success or failure was judged based on the movement duration, with trials having a movement duration less than 2.3 s regarded as successful. To quantify task performance during the test phase and compare training conditions, the success rate was calculated for each block during the test phase. Furthermore, we quantified movement corrections of the cursor trajectories by measuring the number of speed peaks (nSP) and the direction error after the first movement correction (cDE) in each test trial (27). To estimate the nSP, the peaks of the cursor velocity were first determined with MATLAB function *findpeaks*. Since the function might detect even small peaks in cursor speed, the argument “MinPeakProminence” was set to 5% of the maximum speed for each trial. Subsequently, we defined the nSP as the number of peaks divided by the movement duration in each trial. The cDE was calculated as the absolute angle between the direction from the cursor position at the first velocity peak to the target position and the direction from the cursor position at the first velocity peak to the cursor position at the second velocity peak. In some trials, the nSP was either 0 or 1. If the nSP was 0, the trial was excluded from the analysis. If the nSP was 1, the cDE was computed as the absolute angle between the direction from the cursor position at the first velocity peak to the target position and the direction from the cursor position at the first velocity peak to the cursor reach position.

### 2.5 Statistics

To evaluate the differences in the learning and exploration process between training conditions, a generalized linear mixed-effect model analysis was performed. The dependent variables were movement duration, tortuosity, MV, RV, and task-irrelevant variability. The independent variables were block and training conditions. All models included “participant” as the random intercept effect. The dependent variables can only be non-negative values. Thus, the probability distribution was set to a gamma distribution, and a log-link function was used. The equation for this model is as follows:

$$\begin{array}{rl} & \log(y)\;∼\;\mathrm{block}\;+\;\mathrm{condition}\;+\;\mathrm{block}\;\;∙\;\mathrm{condition}\\ & y\;∼\;\mathrm{Gamma} \end{array}$$

If a significant interaction was found, Welch's *t*-test was performed on the difference in mean between training conditions in the same block.

A two-tailed permutation test was then performed to test for differences in success rates between the small and wide target conditions. Furthermore, a 95% confidence interval of the mean difference between the conditions was obtained using the bootstrap method.

To evaluate the difference between training conditions in the performance of the corrective movements in the first block of test trials. Linear mixed-effect model analysis was performed. The dependent variable was any one of nSP and cDE. The independent variables were target and training conditions. This model included “participant” as the random intercept effect. The equation for the model is as follows:

$$\begin{array}{rl} & y\;∼\;\mathrm{target}\;+\;\mathrm{condition}\\ & y\;∼\;\mathrm{Normal} \end{array}$$

Statistical models were constructed to predict success rates during the test phase. We performed a mixed-effects logistic regression analysis. The dependent variable was a dummy (0 = failure, 1 = success). The independent variables were block, target, task-irrelevant variability in the last block of the training phase and MV calculated from all trajectory data during the training phase. This model included “participant” as the random intercept effect. The equation for this model is as follows:

$$\begin{array}{rl} & \mathrm{logit}(y)\;∼\;\mathrm{block}\;+\;\mathrm{target}\;+\;\mathrm{task}\;\mathrm{irrelevant}\;\mathrm{variability}\;+\;\mathrm{MV}\\ & y\;∼\;\mathrm{Bernoulli} \end{array}$$

In all statistical analyses, a *p*-value of less than 0.05 was considered statistically significant.

## 3 Results

### 3.1 Cursor trajectories during the learning process

**Figure 2:**
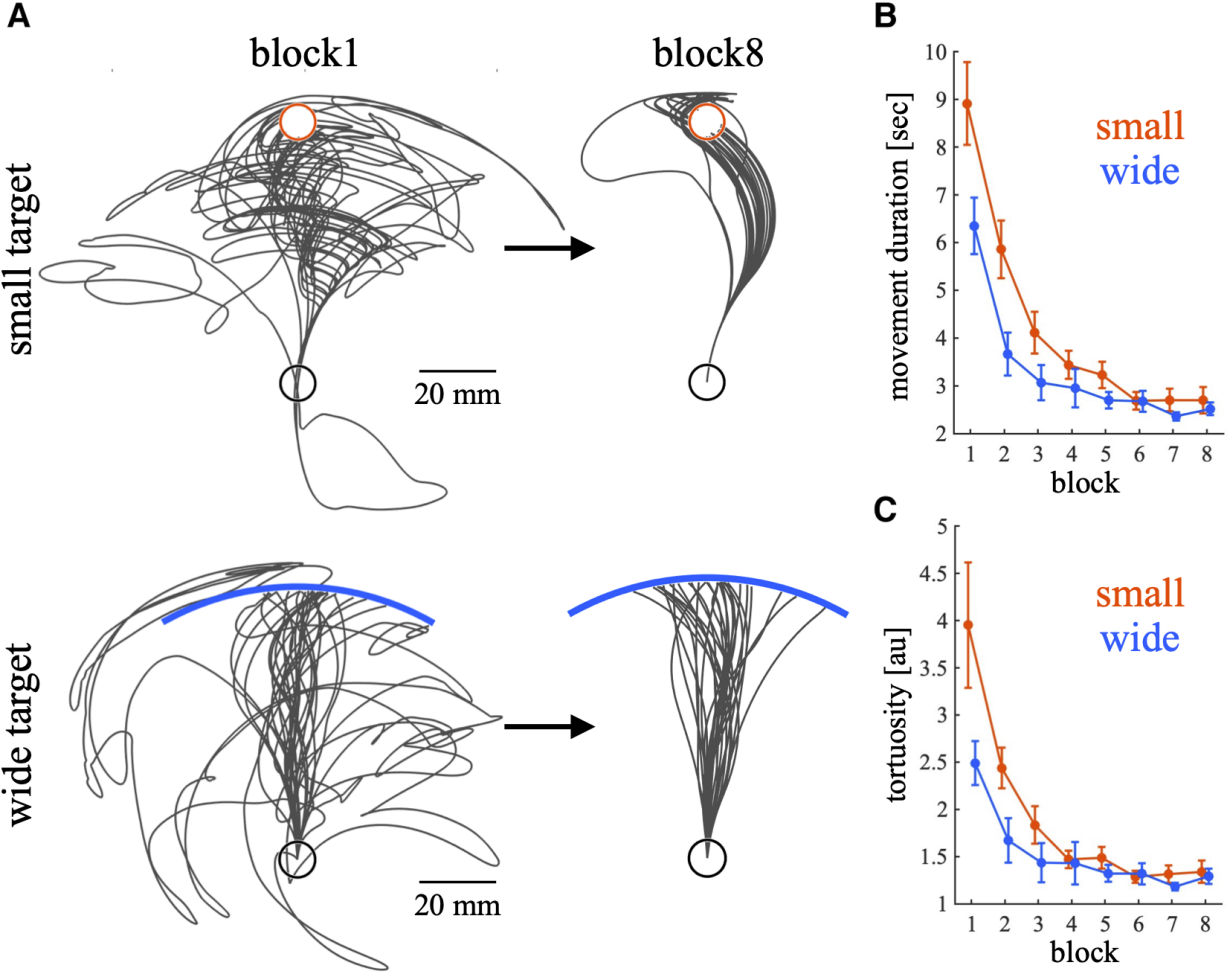
Raw cursor trajectories in blocks 1 and 8, movement duration and tortuosity during the training phase. (**A**) The trajectories of the cursor are illustrated with black lines for two representative participants from small and wide target groups, respectively. Trajectories for both block 1 and block 8 are represented the same participant. The targets for each group are indicated differently: a red circle represents the small target, and a blue bar denotes the wide target. (**B,C**) Movement duration (**B**) and tortuosity (**C**) across trials. Thick lines indicate the mean across participants. The error bars indicate the mean ± standard error across participants. The red and blue lines represent the small and wide target conditions, respectively. When creating figures, data exceeding the 95th percentile (movement duration: 11.185 s; tortuosity: 4.447) were excluded.

Participants performed a *de novo* visuomotor learning task. Figure 2A shows representative cursor trajectories in block1 and block8 during the training phase for each group. During block1 in both conditions, the cursor trajectories deviated significantly from the straight path to the target. However, during block8, the deviation of the cursor was smaller than that of block1. As the trials progressed, the movement duration decreased, indicating that the participants learned to move the cursor faster during the training phase (Figure 2B). Significant main effects were observed in training condition [*p* < 0.01, 95% CI = (−0.69, −0.20); wide—small], block [*p* < 0.01, 95% CI = (−0.18, −0.14)] and the interaction between condition and block [*p* < 0.01, 95% CI = (0.024, 0.084)]. Post hoc t test shows that there are significant differences between training condition in block1 (*p* = 0.022) and block2 (*p* < 0.01). Figure 2C shows the change in tortuosity during the training phase. Participants in both conditions gradually became able to reach the target more directly, whereas tortuosity tended to be higher in the small target condition during the early training phase. Significant main effects were observed in training condition [*p* < 0.01, 95% CI = (−0.72, −0.22); wide – small], block [*p* < 0.01, 95% CI = (−0.16, −0.12)] and the interaction between condition and block [*p* < 0.01, 95% CI = (0.032, 0.092)]. Post hoc t test shows that there are significant differences between training condition in block2 (*p* = 0.023). These results indicate that during the early training phase, the movement duration and tortuosity tended to be higher in the small target condition than in the wide target condition, and little difference was observed between the training conditions during the last training phase. In summary, the participants in both conditions learned to move the cursor to the target quickly and directly.

### 3.2 Difference in the exploration process between small and wide target conditions

**Figure 3:**
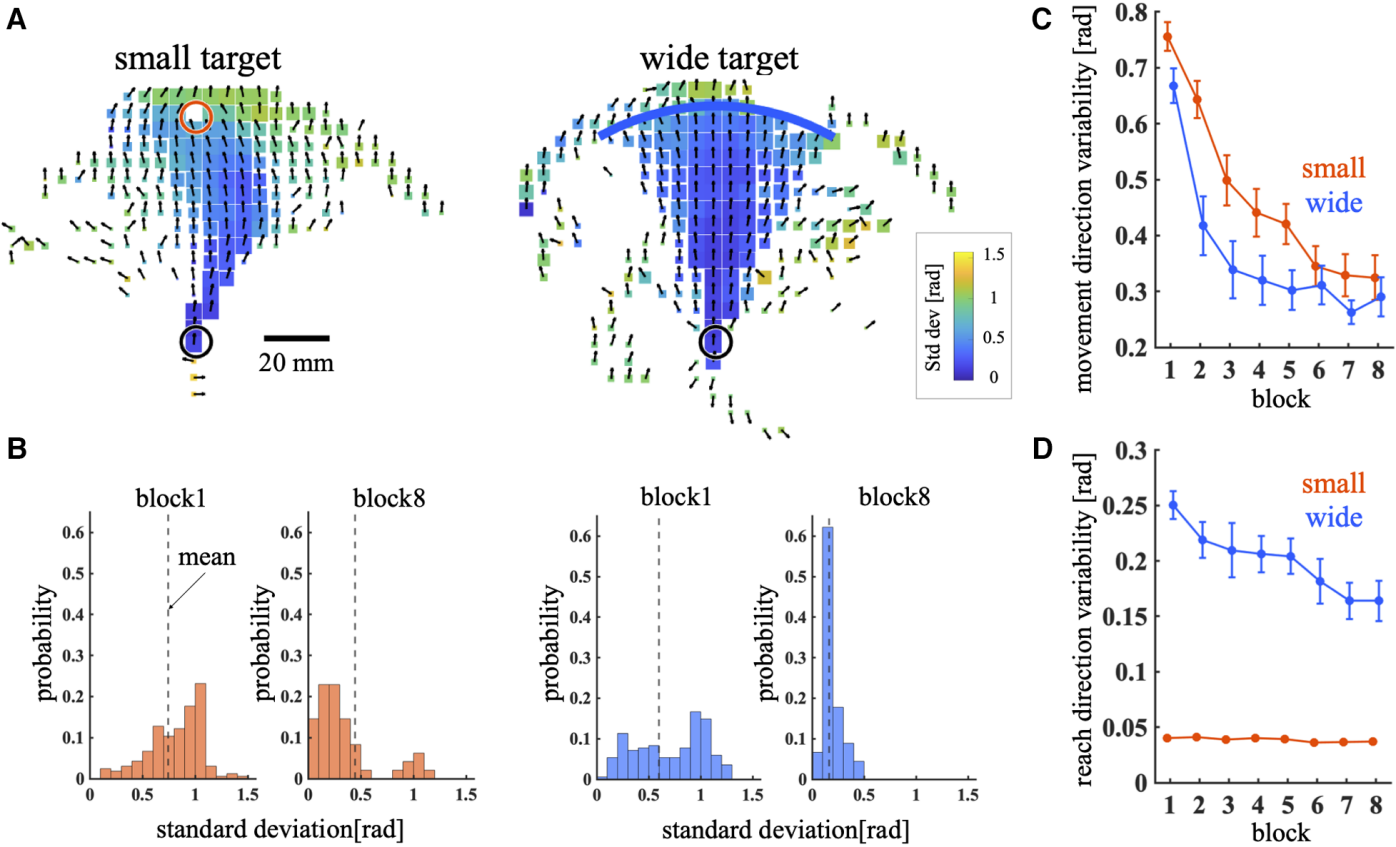
Movement direction variability and reach direction variability. (**A**) Mean and standard deviation of the movement direction across all trajectories during the training phase. The direction of the arrow and color of the bin represent the mean and standard deviation of the movement direction, respectively. The bin size represents the number of times the cursor passes through the bin. (**B**) Histogram of the standard deviations of all bins. The dashed line represents the mean across all bins weighted by the number of visits to each bin. (**C,D**) MV (**C**) and RV (**D**) across blocks. Thick lines indicate the mean across participants. Error bars indicate mean ± standard error across participants. The red and blue lines represent the small and wide target conditions, respectively.

To compare exploration during the learning process between training conditions, two variability indices were quantified: movement direction variability and reach direction variability. The mean ± SD movement angle in each bin of the cursor movement space is shown in Figure 3A. It is apparent that the angular standard deviation of the bins near the target was higher in the small target condition than in the wide target condition. Figure 3B illustrates the distribution of the angular standard deviation for all bins in block1 and block8. This result revealed that the mean distribution decreased as the blocks progressed. The quantitative results throughout all blocks are presented in Figure 3C. Throughout training, MV gradually decreased under both training conditions (Figure 3C). Significant main effects were observed in training condition [*p* < 0.01, 95% CI = (−0.66, −0.097); wide – small] and block [*p* < 0.01, 95% CI = (−0.16, −0.11)]; the MV in the small target condition was higher than that in the wide target condition. The interaction between condition and block was not significant [*p *= 0.17, 95% CI = (−0.01, 0.06)]. Figure 3D shows the RV during the training phase. The RV in the wide target condition was higher than that in the small target condition during the training phase (Figure 3D). The main effect of training condition [*p* < 0.01, 95% CI = (1.65, 2.01); wide – small] and interaction between condition and block [*p* < 0.01, 95% CI = (−0.074, −0.02)] were significant. However, the main effect of the block [*p* = 0.082, 95% CI = (−0.035, 0.002)] was not significant. Post hoc t test shows that there are significant differences between training condition in all blocks (*p* < 0.01 for all blocks). In summary, although the RV was higher in the wide target condition, the MV was higher in the small target condition. These results indicated that exploration during the learning process differed between the small and wide target conditions.

### 3.3 Generalization of *de novo* learning

**Figure 4:**
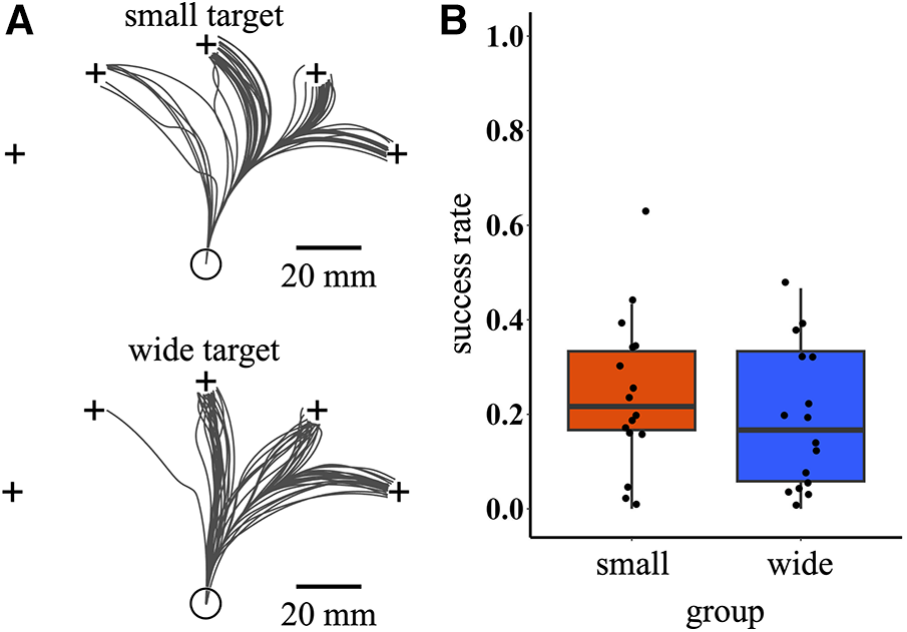
Raw success trajectories and success rate during the test phase. (**A**) Cursor trajectories of all successful trials during the test phase for two representative participants selected from small and wide target conditions, respectively. The black lines indicate cursor trajectories. (**B**) Boxplot of success rate in the first block of the test phase. The circular dots indicate the success rate of each participant.

After training, the participants performed the test task. Figure 4A shows the raw success trajectories of representative participants. The success rate was calculated as an index of the extent to which the participants could manipulate the cursor. The success rates in the first block of the test phase were compared between the two training conditions. Although there was weak trend that the small target condition had a higher success rate [95% bootstrap CI = (−0.16, 0.05); wide – small], no significant difference was observed between training conditions (permutation *p*-value = 0.351). Notably, large individual differences were observed in the success rate, regardless of the training condition (Figure 4B).

### 3.4 Corrective movements for untrained targets

**Figure 5:**
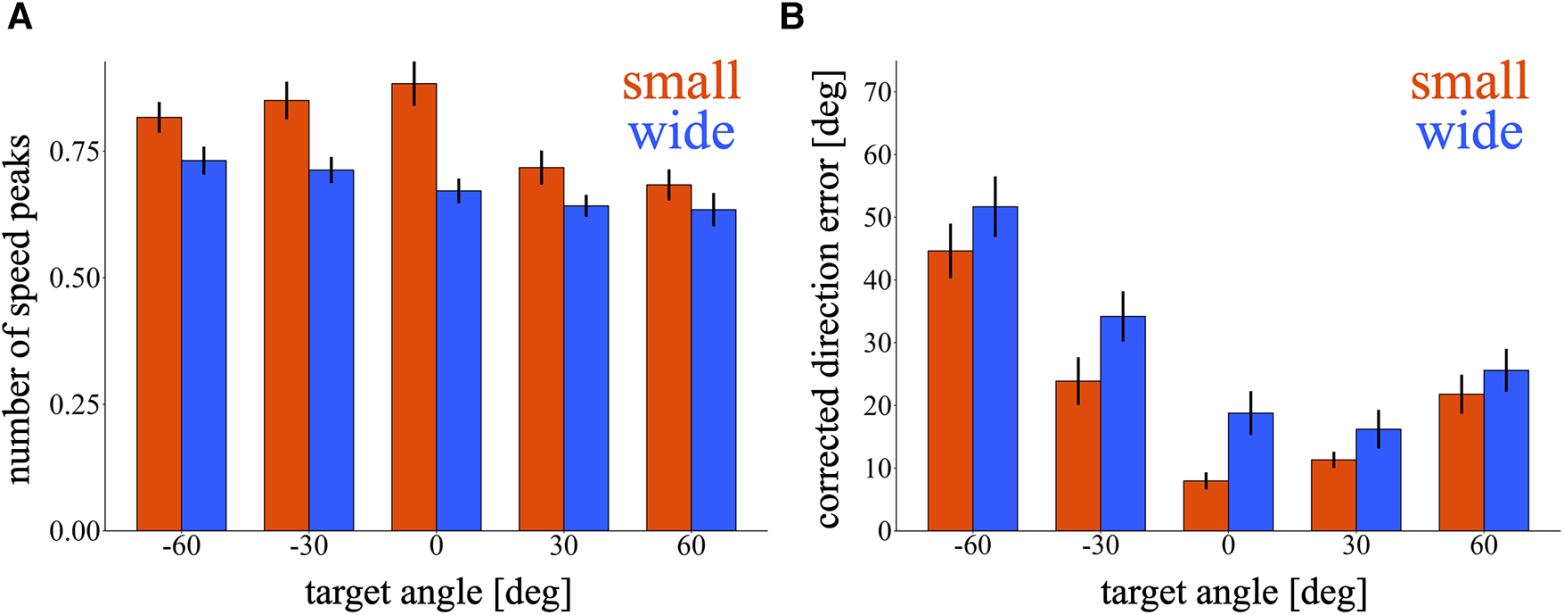
Corrective movements during the test phase. (**A,B**) Bar graph of the number of speed peaks (**A**) and corrected direction error (**B**) in the first block of the test phase. The red and blue bars represent the small and wide target conditions, respectively. Error bars indicate mean ± standard error across participants.

We quantified the corrective movements of cursor trajectories during the test phase. In Figure 5A, the nSP was compared between the small and wide target conditions at each target. It appears that the small target condition exhibited higher nSP for all targets. A marginally significant effect was observed in training condition [*p* = 0.082, 95% CI = (−0.23, 0.01); wide – small], demonstrating that the small target group performed more corrective movements aiming to the test targets. Additionally, a main effect of target was significant [*p* < 0.01, 95% CI = (−0.056, 0.049), (−0.048, 0.056), (−0.15, −0.046) and (−0.17, −0.067); −60°, −30°, 30° and 60° relative to 0°, respectively], indicating that the targets on the right side shows fewer nSP. Figure 5B shows the cDE calculated in both the small and wide target conditions at each target. The small target group seems to have lower cDE for all targets. We observed a marginally significant effect in the training condition [*p* = 0.08, 95% CI = (−0.59, 15.4); wide – small], suggesting that the small target group exhibited better movement correction towards the test targets. A main effect of target was significant [*p* < 0.01, 95% CI = (28, 41), (9.2, 22), (−6.0, 6.8) and (3.9, 17); −60°, −30°, 30° and 60° relative to 0°, respectively], indicating that the cDE increased as the target direction was away from the 0° direction. In summary, the small target group demonstrated more and better movement correction aiming towards the test targets.

### 3.5 Task-irrelevant control space variability related to generalization of *de novo* learning

**Figure 6:**
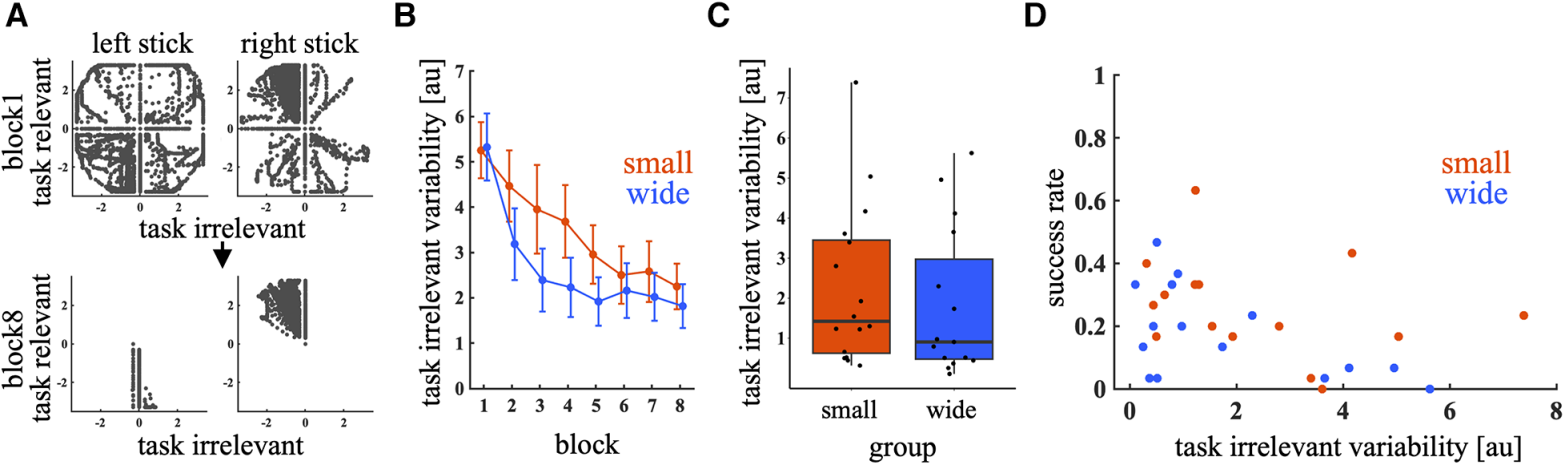
Variability in joystick input. (**A**) Representative joystick inputs in blocks 1 and 8 during the training phase. The black dots represent joystick inputs. (**B**): Task-irrelevant variability across blocks. Thick lines indicate the mean across participants. Error bars indicate mean ± standard error across participants. The red and blue lines represent the small and wide target conditions, respectively. (**C**) Box plot of task-irrelevant variability in the last block of the training phase. Circular dots indicate the task-irrelevant variability of each participant. (**D**) Relationship between task-irrelevant variability in the last block of the training phase and the success rate in the first block of the test phase. The red and blue dots represent the small and wide target conditions, respectively.

Large individual differences in success rates in the test trials were examined. To investigate the factors causing individual differences, the learning process in a joystick control space was analyzed. Figure 6A shows the joystick inputs during the first and last blocks of the training phase. In the first block, the input was broadly distributed in both task-relevant and task-irrelevant directions. However, in the last block, the inputs converged in the task-relevant space. Figure 6B shows that the task-irrelevant variability decreased under both training conditions. A significant main effect was observed in block [*p* < 0.01, 95% CI = (−0.20, −0.11)]. The main effect of training condition [*p* = 0.38, 95% CI = (−0.73, 0.28); wide – small] and interaction between condition and block were not significant [*p *= 0.72, 95% CI = (−0.076, 0.053)]. Although the task-irrelevant variability decreased, it varied among individuals in the last block (Figure 6C). A mixed-effects logistic regression model analysis shows the significant effect of the task-irrelevant variability on success rate [*p* = 0.011, 95% CI = (−0.61, −0.079)], indicating that participants with high task-irrelevant variability at the end of the training phase tended to have a low success rate in the test phase (Figure 6D). On the other hand, no significant effect of MV was observed [*p* = 0.50, 95% CI = (−2.6, 5.3)]. The main effect of block [*p* < 0.01, 95% CI = (0.30, 0.46)] and target was also significant [*p* < 0.01, 95% CI = (−5.4, −4.2), (−2.5, −2.0), (−0.24, −0.23) and (−1.5, −1.04); −60°, −30°, 30° and 60° relative to 0°, respectively], indicating that the success rate increased as the block progressed, and it decreased as the target direction was away from the 0° direction.

## 4 Discussion

This study aimed to clarify how the exploration process in *de novo* learning affects the generalization of newly acquired skills. To manipulate the exploration process, *de novo* learning tasks were conducted in two groups: one group controlled the cursor to hit a small target, while the other aimed at a wider target. The results revealed that participants in both groups learned to move the cursor to the target quickly and directly (Figures 3A,B), whereas the exploration process varied between the groups (Figures 4C,D). However, no significant differences were observed in the success rates of the test blocks as a generalization measure between the groups. Remarkably, we found large individual differences in success rates (Figure 4B). A statistical model explaining this individual difference indicates that participants with high task-irrelevant variability at the end of the training phase tended to have low success rates in the test phase.

During the test phase, more than half of the participants in the small target condition achieved a success rate above 0.2 (Figure 4B), indicating that they could succeed in directions they had not trained to during the training phase, that is, directions other than 0°. This result suggests that generalization occurs during *de novo* learning. However, a notable observation was the large individual difference in success rate, implying that the extent of generalization varies, even with the practice of aiming at an identical target.

Our study focused primarily on how the generalization of newly acquired skills is affected by the exploration process in *de novo* learning tasks with small and wide targets. These tasks are characterized by motor exploration involving corrective movements and various reaching directions. Indeed, the MV was consistently higher in the small target condition throughout the training phase (Figure 3C), suggesting that a diverse range of corrective responses to the target depends on the state of the cursor. Conversely, the RV was predominant in the wide target condition (Figure 3D). It was possible that the RV of the wide target condition decreased over the course of the training and became as high as that of the small target condition. However, the difference in the RV between the small and wide target conditions was prominent throughout the entire training phase (Figure 3D). This result suggests that the participants in the wide target condition exploited the exploration strategy of reaching in a broader direction rather than finding a reach direction that they were good at and repeating that movement. A comparison of the generalizations between these two target conditions revealed a slight tendency for participants in the small target condition to achieve higher success rates than those in the wide target condition (Figure 4). These results can be interpreted from a motor control perspective. The tasks in this study required movements lasting over 2 s (Figure 2B) with continuous visual feedback from the cursor provided during the task. In such situations, feedback control is crucial for correcting online movement errors, which depend on sensory information with a time delay (28). Furthermore, a previous study reported that skilled learning affects feedback control (29). Specifically, the small target condition, compared with the wide target condition, requires the acquisition of more precise online feedback corrections. The higher MV observed in the small target condition likely reflects increased opportunities for online feedback corrections (Figures 3A,C). Furthermore, a greater number of movement corrections were observed in the cursor movements with more precise targeting to the test targets in small target conditions (Figures 5A,B). Therefore, the slight trend toward stronger generalization in the small target condition could be attributed to enhanced learning in the feedback controller.

Previous studies have demonstrated that patterns of generalization are formed around actual rather than planned movements (14, 15). Based on these findings, one might expect that an increase in the variability of the actual movements could enhance generalization. This perspective offers an additional interpretation for our observation that participants in the small target condition achieved slightly higher success rates. The participants in the small target condition could only reach the target in the 0° direction. In contrast, the participants in the wide target condition could reach a broader range of directions (Figure 3D). Given the difference in the reachable range, one could predict greater generalization in the wide target condition. However, contrary to the prediction, there was no significant difference in generalization; instead, generalization tended to be larger in the small target condition. A notable result during the training phase was that participants in the small target condition experienced a more diverse range of movement directions than those in the wide target condition (Figure 4C). These results suggest that not only the variability in the reach direction, but also the variability in the movement direction during the middle of the actual movement, may have encouraged further exploration and facilitated generalization.

The relationship between motor variability and the rate of motor learning has been previously reported (17–20). Consequently, motor variability is a crucial factor when considering the individual differences in motor learning. In this study, we specifically examined the task-irrelevant motor variability in the joystick control space. Statistical model analysis revealed that participants exhibiting high task-irrelevant variability at the end of the training phase tended to have low success rates in the test phase. The role of task-irrelevant variability in *de novo* motor learning was explored in a previous study using a mathematical model (23). The authors theoretically proved that eliminating task-irrelevant components through extensive exploration in entire motor command space facilitated error corrections during entire learning phase. Our results that joystick inputs converged in the task-relevant space is consistent with the previous theory (Figure 6A). Furthermore, this study demonstrated that the participants with lower task-irrelevant variability at the end of training exhibited higher success rates in the test phase (Figure 6D). These findings imply that while task-irrelevant variability can enhance error corrections during learning, its reduction in the final training stages is crucial for better generalization of *de novo* learning. However, future research is necessary to further understand how variability in control space affects the relationship between error correction and generalization in *de novo* learning due to differences in experimental setups between this and previous studies. Specifically, the previous study used a simulation of a feedforward motor task, such as shooting task, whereas our study involved a motor task that required online feedback correction.

In the test phase, participants had more success when the targets were on the right side than on the left (Figure 4A). This result can be attributed to the anisotropic nature of the manipulability ellipsoid in the two-link arm system. In our experimental setting, the forward diagonal direction to the right exhibited a higher manipulability from the start position. This led to differences in success rates among the five test targets. Future research should focus on exploring the spatial generalization patterns in *de novo* learning, potentially using methodologies that address these constraints.

In conclusion, this study examined the generalization of *de novo* visuomotor learning tasks by focusing on the impact of different target shapes. The results demonstrated that, while generalization occurred in *de novo* learning, it was more closely associated with task-irrelevant variability in the control space than with the target shapes. These findings underscore the significance of exploratory behavior within the control space as a driving force for the generalization of *de novo* learning. Such insights could have important implications for designing more effective learning environments that can leverage generalization.

## Data Availability

The original contributions presented in the study are included in the article, further inquiries can be directed to the corresponding author.
